# News and Notes

**Published:** 1903-12

**Authors:** 


### NEWS AND NOTES.

IT will be of interest to all physicians in the State to learn that a professional nurses' register has been established at Palmer's Viaduct Pharmacy, 11 Peachtree street, Atlanta. Only graduates and competent names are on this register, and one can be obtained by either writing, telegraphing or telephoning them. Phone 64.

---

AT the 29th Annual Session of the Mississippi Valley Medical Association held at Memphis, October 7–9, the following resolutions were adopted :

In view of the fact that more than 400 deaths from Tetanus occurred following the 4th of July celebration of 1903, as shown by the statistical report elaborated by Dr. S. C. Stanton, of Chicago, and published in the Journal of the American Medical Association of August 29, 1903, the great majority of which might have been prevented had proper precautions been taken ; therefore

*Be it Resolved*, That the conclusions which follow, as offered by Dr. Stanton in a paper presented before the Association, at the above meeting, be endorsed as the sense of the Association, and further

*Be it Resolved*, That the Secretary be instructed to forward a copy of these resolutions and conclusions to the Medical Press, Associated Press, and the Secretaries of the several State Medical Societies, with the request that they publish same and take suitable action thereon.

1. Enforcement of existing laws regarding the sale of Toy Pistols and other dangerous toys.
2. Enactment of laws by the nation, States and municipalities prohibiting the manufacture and sale of Toy Pistols, Blank Cartridges, Dynamite Canes and Caps, Cannon Crackers, etc.
3. Open treatment of all wounds, however insignificant, in which from the nature or environment there is any risk of Tetanus.
4. Immediate use of Tetanus Antitoxin in all cases of Fourth-of-July wounds, or wounds received in barnyards, gardens, or other places where Tetanus infection is likely to occur.
5. As a forlorn hope, the injection of Tetanus Antitoxin after Tetanus symptoms have appeared.

### MORE LIGHT ON TUBERCULOSIS.

——— GA., September 30, 1903.

——————

I gave your son a turough examination to-day, I find NO tuberculosis at all,

I find that he has had a light attact Dengue fever within the last two months, It is a form of malarial, and some times it leaves a trouble behind that is hard to cure, the disease its self it never fatal, hardly ever stoping one from work, but it leaves them very feble, the hemorage that he had was from the broncal tube, While creasote is a very good in such cases, yet it alone will never cure him,

Giving creasote in such cases reminds me of a man that tries to fatten his horse, on grass a lone, then the creasote treatment is out of date.

Mrs. ——— was taking it in 12 drop doses when she came to me, I stoped it at once, my rule is give only the best of medicines. Your son can not be cured without medicined. and that is large quantities. a change of climent is no good in his case.

the trouble with your doctors is two many cases, and two little attention given to any one, doctors in citys over croud there practis, the man that cures you boy will have somthing to do besides talk, and you boy will have to track the treatment to the letter, in other words it would be necessary to have some one to give the medicines to him, as they are marked on the bulitin, he could not be trusted to take it at all, a thing that is worth doing is worth doing well,

From what he told me, I am satisfied that his wife had a rent in the vagina, that set up vaginitis and it was let run untill it got in to her blood and the lung being the weakest point, it set up the abseses in the lungs, that caried her off, a number of women die that way ever year, it has got to be so comon that it now has taken the name of VAGINAL CONSUMPTION.

Yet the doctors that have never investigated the mater, hoot at it, yet the same doctor will admit that an inflamed overy, will effect a womans head & cause her to go to the asilum.

he told me that one Dr. told that it came from the vaginal trouble but that he did not believe him, & got another Dr.

one other thing that I see in his case, that is that he has ben told and led to believe that he has consumption, and you can not cure a man a gainst his will,. he told to me that he cought it from his wife, I ask him if any one else cought it from her he said, that his Dr. said that a man has to be between the age of 15. & 30. to ketch it, said it was infectious, now in fact and vacinate is one and the same, So I say and prove by the records that any disease that has pus or matter is infectious, O dont suppose that he took any of the matter from her and put it under his skin, if he did he would have died before she did,

Had he have had her vagina treated after she had that child she would have never had that cough, She died from one form of blood poisen, and the first thing I done for Mrs. H. when she arrived here, was to cure up that bad vaginitis, that was the cause of her cough. she said that no Dr. has ever as much as ask her about it, but all said she had consumption.

I gave B. some medicines, merely to see if it was correct in my diagnosis,

And I did not say any thing to him about treatment, as he said he was only to stay a few days.

